# Seroprevalence and Associated Factors of Anti-*Strongyloides* spp. IgG Among Primary School Children in Southern Thailand

**DOI:** 10.3390/pathogens15060566

**Published:** 2026-05-24

**Authors:** Prasit Na-Ek, Udomsak Narkkul, Nonthapan Phasuk, Stephen J. Scholand, Chuchard Punsawad

**Affiliations:** 1 School of Medicine, Walailak University, Nakhon Si Thammarat 80160, Thailand; prasit.na@wu.ac.th (P.N.-E.); udomsak.na@wu.ac.th (U.N.); nonthapan.ph@wu.ac.th (N.P.); 2 Center of Excellence in Tropical Pathobiology, Walailak University, Nakhon Si Thammarat 80160, Thailand; 3 Department of Medicine, University of Arizona, Tucson, AZ 85724, USA; rabiesfreeworld@yahoo.com

**Keywords:** *Strongyloides stercoralis*, seroprevalence, primary school children, Thailand

## Abstract

*Strongyloides stercoralis (S. stercoralis)* is an important soil-transmitted helminth that infests over 600 million people worldwide. However, data on its seroprevalence in remote regions, such as Thailand’s island areas, remain limited. This study examined the seroprevalence and associated risk factors of anti-*Strongyloides* spp. IgG seropositivity among primary school children in Koh Yao, an island in southern Thailand. A total of 351 primary school children (156 males and 195 females) were included. The seroprevalence of anti-*Strongyloides* spp. IgG was determined using the *Strongyloides*-specific IgG antibodies ELISA, and risk factor data were collected through a questionnaire. Hematological parameters were also analyzed. Univariate and multivariate logistic regression analyses were used to assess associations between risk factors and anti-*Strongyloides* spp. IgG seropositivity. The seroprevalence of anti-*Strongyloides* spp. IgG was 3.7% (13/351 participants). Analysis of the risk factors revealed that participants who drank filtered water exhibited lower odds of anti-*Strongyloides* spp. IgG seropositivity compared to those who drank tap or rainwater (AOR = 0.21, 95% CI 0.05–0.95, *p* = 0.043). However, due to the small number of seropositive cases, this association is hypothesis-generating and likely serves as a proxy for better household hygiene rather than a direct protective factor. This study is the first report on anti-*Strongyloides* spp. IgG seropositivity among primary school children in Koh Yao, southern Thailand, demonstrating a low seropositivity rate in this population. These findings provide location-specific information on modifiable risk behaviors, aiding in developing more effective control and prevention strategies for anti-*Strongyloides* spp. IgG seropositivity in Thailand’s island area.

## 1. Introduction

Human strongyloidiasis is caused by a roundworm parasite from the genus *Strongyloides*, specifically *S*. *stercoralis*, which is widely distributed in tropical and subtropical regions worldwide, including Thailand [1,2,3]. However, another species, *S. fuelleborni*, has also been reported to infect humans in certain geographical areas, particularly where humans live in close proximity to non-human primates [4]. *S*. *stercoralis* is estimated to infect more than 600 million people worldwide, and many cases remain undetected [5,6]. Strongyloidiasis can cause symptoms primarily affecting the intestines, lungs, and skin. Individuals with asymptomatic infections may harbor the parasite for years [2]. Although strongyloidiasis symptoms are typically mild, immunocompromised individuals may develop severe or fatal infections due to disseminated strongyloidiasis and hyperinfection, with mortality rates of approximately 68.5% and 60%, respectively [7]. The southeastern region of Asia is considered a hotspot for strongyloidiasis, with prevalence rates higher than the global average [6,8]. In Thailand, prevalence ranges from 0.05% to 79.6%, with a pooled *S*. *stercoralis* prevalence of 11.3%—slightly lower than Southeast Asia’s overall pooled prevalence of 12.7% [3,8,9,10,11]. Data from Thailand’s four regions (Central, Northern, Northeastern, and Southern) indicate the highest average *S*. *stercoralis* prevalence in the northeastern region (22.5%), followed by the North (15%), South (8.3%) and Central (6.6%) regions [8].

*S. stercoralis* infection is typically acquired through skin penetration by infective filariform larvae present in contaminated soil. Risk factors for transmission include walking barefoot, poor sanitation, direct contact with contaminated soil or water, and exposure to animal reservoirs. In Thailand, the diversity of climate, topography, and human activities contributes to wide regional variation in prevalence. Rural agricultural communities, particularly in the northeastern and southern regions, exhibit higher infection rates due to frequent soil contact and limited access to safe water and sanitation, whereas urbanized areas report relatively lower rates [9,12,13]. These context-specific differences suggest that environmental and behavioral factors together drive the heterogeneous distribution of strongyloidiasis across the country. Over the past two decades, Thailand has implemented national parasitic disease control programs, primarily targeting soil-transmitted helminthiases and lymphatic filariasis [14,15]. The Ministry of Public Health introduced national deworming campaigns in school-aged children in alignment with the World Health Organization’s roadmap, using annual or biannual mass drug administration (MDA) with albendazole or mebendazole [14]. These interventions have substantially reduced the prevalence of common intestinal helminths but may have had limited impact on *S. stercoralis*, as benzimidazoles exhibit lower efficacy against this parasite [16]. In parallel, Thailand achieved elimination of lymphatic filariasis as a public health problem in 2017 after more than a decade of MDA programs distributing albendazole combined with diethylcarbamazine (DEC) or ivermectin [15], which likely contributed to reduced *S. stercoralis* transmission in some regions. Such large-scale antiparasitic interventions may therefore explain regional variations in the distribution and prevalence of strongyloidiasis across the country.

However, studies on people from southern Thailand have reported prevalence rates ranging from approximately 0.8% to 40% [3,8,17]. Most studies in southern Thailand have relied on coprological methods, potentially underestimating *S*. *stercoralis* prevalence [3,18]. These studies have primarily focused on soil-transmitted and zoonotic helminths in mainland areas, leaving a knowledge gap on strongyloidiasis seroprevalence when using highly sensitive and specific serological methods, particularly in southern Thailand’s islands [3,8,19,20,21]. We conducted a survey in the island areas of the Phang Nga Province using serological methods to determine the seroprevalence of anti-*Strongyloides* spp. IgG in schoolchildren, addressing this gap. Primary school children were selected as the target population because this group is highly susceptible to *S. stercoralis* and other soil-transmitted helminths due to frequent outdoor play, direct contact with soil, and ongoing development of personal hygiene behaviors. Focusing on this age group allows for the assessment of transmission dynamics and facilitates targeted intervention strategies [22]. In addition, this study investigated the hematological parameters and potential risk factors associated with anti-*Strongyloides* spp. IgG seropositivity among primary school children residing in the Koh Yao Island area of Phang Nga Province.

## 2. Materials and Methods

### 2.1. Study Participants, Setting, and Enrollment Criteria

**Figure 1:**
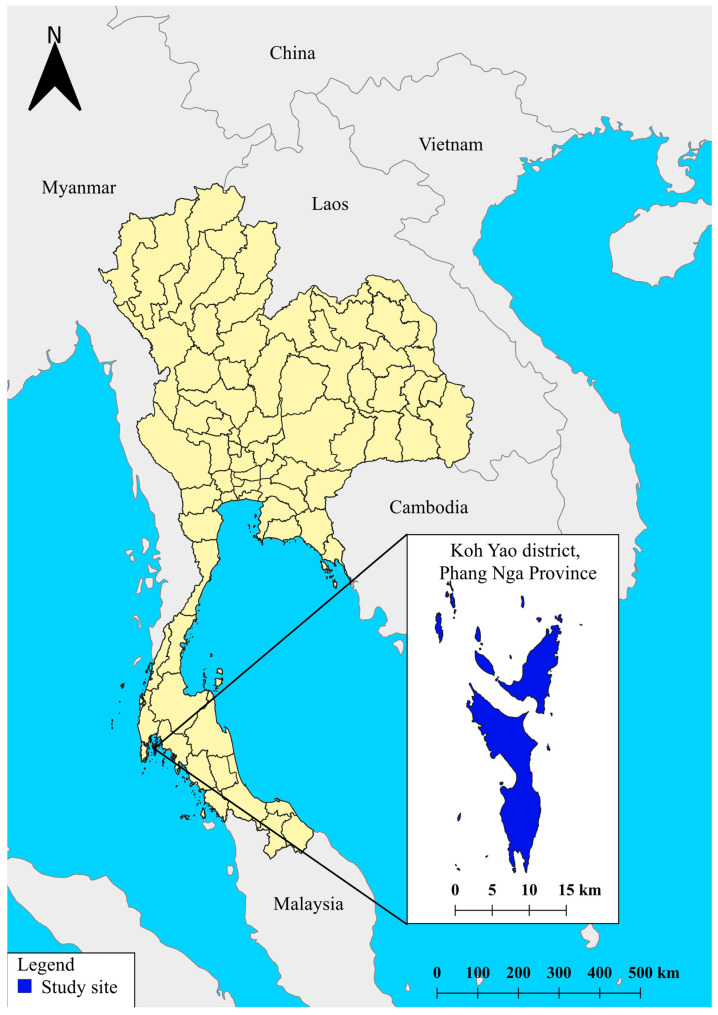
Map illustrating the study area in Koh Yao District, Phang Nga Province, Thailand. Quantum GIS version 3.16.11 (with ESRI base maps) was used to create the map.

A cross-sectional study design was used, and all eligible primary school children aged 7–12 years within the selected schools in Koh Yao district, Phang Nga Province (Figure 1) were invited to participate (total population sampling). No random sampling was applied; instead, an exhaustive sampling frame was established for all children meeting the inclusion criteria. The field data collection and blood sampling were conducted from 9 January to 30 May 2023, corresponding to the dry season in southern Thailand. This period was selected to ensure continuous school attendance and facilitate access to all island schools. According to the Phang Nga Primary Educational Service Area Office, the district comprises 12 government schools with a total student population of 1279—666 males and 613 females. The inclusion criteria for the study required participants to be children aged 7–12 years of either sex who were enrolled in one of the 12 government schools in Koh Yao District. Recruitment of participants was carried out through collaboration with school administrators and teachers. Study information sheets and parental consent forms were sent home with all eligible students, describing the study objectives, confidentiality policies, and procedures for voluntary participation. Parents or guardians who agreed signed the consent forms and returned them to the teachers, after which their children were included in the study following written child assent from each child. This process ensured voluntary participation and compliance with ethical guidelines. The study protocol was approved by the institutional review board. Children with chronic illnesses related to immune system dysfunction, such as human immunodeficiency virus infection or congenital immunodeficiency disorders; those experiencing acute illness with symptoms such as fever during the study period; and those who had received immunosuppressive medications, including corticosteroids, within the previous three months, which were assessed through a confidential, parent-reported health questionnaire without accessing medical records, were excluded. Consequently, participants were comprehensively recruited from all 12 primary schools without any randomization. Participants who declined to participate after initial recruitment within the sample frame were classified as nonrespondents, not exclusions. Exclusion was reserved for individuals who were ineligible prior to recruitment according to the predefined eligibility criteria.

### 2.2. Ethics Approval and Consent to Participate

This study involved human participants and adhered to the guidelines and regulations outlined in the Declaration of Helsinki. This study was reviewed and approved by the Human Research Ethics Committee of Walailak University, Thailand (approval number: WUEC-22-351-01). Written child assent and written informed consent from parents or legal guardians were obtained from all participants for the publication of this paper. Furthermore, parents or guardians of seropositive children were confidentially notified of the results and advised to seek medical follow-up at the local hospital for confirmatory stool examination and appropriate treatment.

### 2.3. Sample Size Calculation

The sample size was calculated using reference data from Eamudomkarn et al., who reported an *S*. *stercoralis* seroprevalence rate of 65.8% among 149 individuals in northeastern Thailand [23]. Although that study targeted adults, it was used because it represented the only available and relevant seroprevalence data for *S. stercoralis* using ELISA in Thailand at the time of study design. Therefore, its reported seroprevalence was considered the most appropriate estimate for calculating the minimum sample size in the absence of comparable data from children. The seroprevalence rate was used as the estimated proportion (*p* = 0.658) in the sample size calculation formula.

$$N=\frac{{Z^{2}}_{\alpha /2}\times PQ}{d^{2}}$$

*α* = 0.05, *Z_α/2_* = 1.96, *P* = 0.658, *Q* = 1 − *P* = 0.342, *d =* 0.05.

The sample size of 346 participants was calculated. After adjusting for a 5% dropout rate, the final sample size was 365.

### 2.4. Questionnaire Survey

This study employed a questionnaire as the primary data collection instrument to obtain information on associated risk factors related to anti-*Strongyloides* spp. IgG seropositivity. Parasitology researchers developed the questionnaire based on data collection and a comprehensive literature review. The questionnaire consisted of three sections: general demographic information, personal health-related data, and behaviors potentially associated with an elevated risk of anti-*Strongyloides* spp. IgG seropositivity. Responses were recorded on a three-point Likert scale: “always,” “sometimes,” and “never.” The questionnaire was subjected to content validation by three experts in pediatric infectious diseases, laboratory medicine and tropical medicine and was pilot-tested with a sample of 30 individuals. It demonstrated good internal consistency with a Cronbach’s alpha coefficient of 0.85. Additionally, the questionnaire was interviewer-administered by trained research staff during school visits. All items were read aloud, and clarifications were provided as needed to ensure understanding and accuracy of responses.

### 2.5. Blood Collection and Preparation

Five milliliters of blood were drawn from the antecubital vein of each subject. Three milliliters were promptly transferred into clotted blood tubes to measure anti-*Strongyloides* spp. IgG antibodies. The samples were stored at 4 °C before being delivered to the medical laboratory for serum separation. Serum samples were stored at −80 °C until further testing. An additional two milliliters of blood was collected in an EDTA tube and stored at 4 °C until delivery to the laboratory for hematological parameter analysis.

### 2.6. Detection of Anti-Strongyloides IgG Antibodies

A *Strongyloides* IgG/IgM ELISA kit (Abcam, Cambridge, UK) was used to measure the IgG antibodies against *Strongyloides* spp. The test had a specificity of 94.12% (95% confidence interval: 83.76–98.77%) and a sensitivity of 89.47% (95% confidence interval: 75.2–97.06%) (Abcam, Cambridge, UK). Serum samples were diluted at a 1:100 ratio using the provided sample diluent, and absorbance was measured at 450/620 nm within 30 s after adding the stop solution. For result interpretation according to the manufacturer’s instructions, samples with absorbance values of less than 9 units were considered negative, whereas those with values greater than 11 units were considered positive, and each sample was tested in duplicate. Equivocal results (absorbance between 9 and 11 units) were re-tested in duplicate; if they remained equivocal, they were conservatively classified as negative to ensure high specificity. The test can cross-react with antibodies against other worms and parasites. However, other soil-transmitted helminths were not examined concurrently in this survey because the study was specifically designed as a seroepidemiological assessment of anti-*Strongyloides* spp. IgG, representing the first serological dataset from Thailand’s island populations. Stool-based copromicroscopic or molecular analyses for other soil-transmitted helminths were beyond the scope of the current ethical approval and field logistics, which focused exclusively on venous blood collection. Moreover, recent parasitological surveys conducted in southern Thailand, including Koh Yao and nearby districts, have already reported updated prevalence data for common intestinal helminths [18].

### 2.7. Investigation of Hematological Parameters

Assessment of hematological parameters was performed using a complete blood count (CBC) test, which included hematocrit (HCT), red blood cells (RBCs), hemoglobin (Hb), mean corpuscular hemoglobin (MCH), mean cell volume (MCV), red cell distribution width (RDW), mean corpuscular hemoglobin concentration (MCHC), platelets (PLTs), white blood cell (WBC) count, and WBC differentiation, including lymphocytes, neutrophils, eosinophils, basophils, and monocytes. These parameters were analyzed using a hematology analyzer (Sysmex XN-330, Kobe, Japan).

### 2.8. Statistical Analysis

The SPSS software (version 17.0; SPSS Inc., Chicago, IL, USA) was used for data entry, cleaning, and analysis. Means and frequencies (percentages) were used to describe the quantitative data. The association between risk factors and anti-*Strongyloides* spp. IgG seropositivity was assessed using univariate logistic regression (ULR) models. In this model, the outcome variable was anti-*Strongyloides* spp. IgG seropositivity, coded as 1 for ELISA-positive and 0 for ELISA-negative participants. Predictor variables (independent variables) included demographic and behavioral factors found relevant in univariate logistic analysis: age group, sex, source of drinking water, use of footwear, handwashing before meals, type of sanitation, and history of anthelmintic use. The multivariate logistic regression model included all potential confounding factors and risk behaviors that have been identified as relevant to anti-*Strongyloides* spp. IgG seropositivity in previous studies, rather than restricting inclusion to variables with *p* < 0.20 from univariate analyses. This inclusive approach was adopted to ensure comprehensive adjustment for confounders and literature-supported risk predictors. We recognize that, although the number of outcome events was relatively small, possibly leading to a low events-per-variable (EPV) ratio, the model was constructed based on biological plausibility and epidemiological relevance. Given this low EPV ratio, the multivariable analysis was treated as strictly exploratory and hypothesis-generating. Differences were considered statistically significant at *p* < 0.05. For categorical variables, reference categories were assigned to represent non-exposed or baseline groups (e.g., male, no anthelmintic use, unfiltered water source). Potential confounders such as age and sex were included in the model a priori based on biological plausibility and the previous literature to control for their influence on anti-*Strongyloides* spp. IgG seropositivity. The results from the ULR and MLR were reported as odds ratios (ORs) and adjusted odds ratios (AORs) respectively. Continuous variables were tested for normality using the Shapiro–Wilk test. Non-normally distributed hematological parameters are summarized as median values (IQR), and the Mann–Whitney U test was used for comparisons between seropositive and seronegative anti-*Strongyloides* spp. IgG individuals.

## 3. Results

### 3.1. Characteristics of Study Participants

The response rate was 96.1%, with 351 out of 365 individuals in the target population responding. There were more female than male participants, and the mean age of the study group was 10 years (range: 7–12 years). Among primary school students, the highest participation rate was observed in 6th-grade students (35.3%). Most study participants reported raising domestic animals (59.83%). Additionally, 51.28% of participants had used anthelmintics within the past year. Their parents primarily worked in non-agricultural sectors (92.31%), such as general employment, merchant/business ownership, and government positions. Regarding personal behavior, most participants washed their hands before meals (98.58%) and after playing (94.59%). In terms of consumption habits, they preferred drinking filtered water (91.17%), boiling or filtering water (88.60%), and avoided eating raw food (59.26%). Most wore shoes when going outside or playing (92.88%) and preferred playing on dirt or grass (97.15%) (Table 1).

### 3.2. Seroprevalence of Anti-Strongyloides spp. IgG

**Figure 2:**
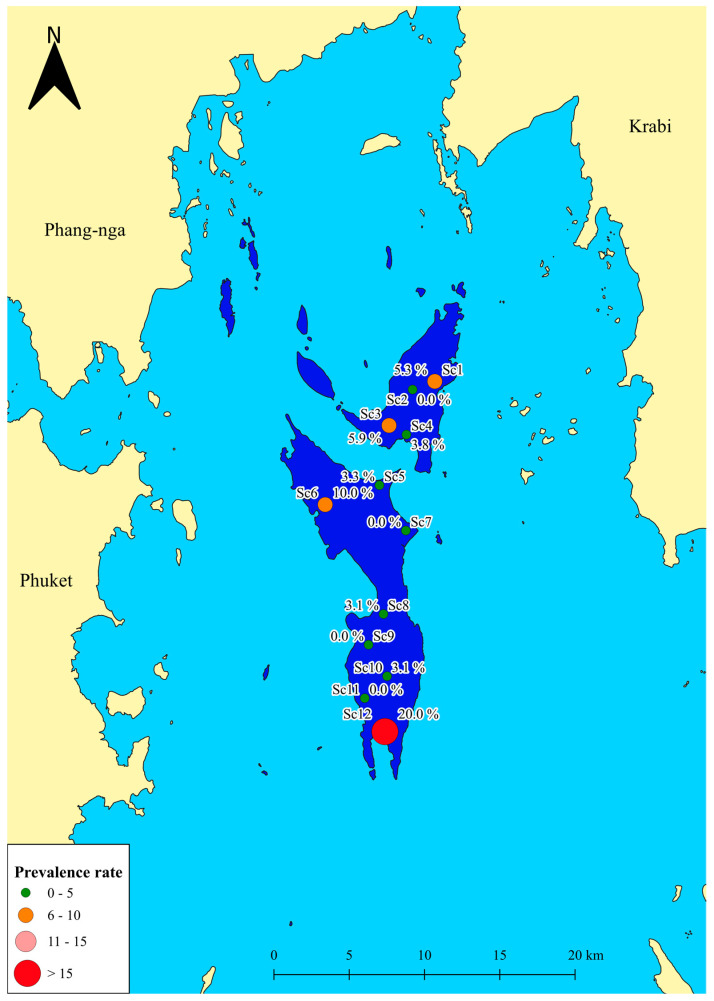
Map of the study sites illustrating the seroprevalence rates of 12 government schools in Koh Yao District. Quantum GIS version 3.16.11 (with ESRI base maps) was used to create the map.

Based on positive serology, the seroprevalence of anti-*Strongyloides* spp. IgG among participants from 12 public schools in the Koh Yao District was 3.7% (13/351). Across schools, the highest seroprevalence was 20.0%, and the lowest was 0%. The results for the remaining schools are summarized in Table 2. Among the subdistricts, Koh Yao Noi had the highest seroprevalence (4.3%), followed by Phru Nai (3.6%) and Koh Yao Yai (3.0%) subdistricts. However, Table 2 and Figure 2 show that the overall differences in the subdistrict seroprevalence rates were not statistically significant.

### 3.3. Factors Associated with Anti-Strongyloides spp. IgG Seropositivity

Among the examined sociodemographic and behavioral variables, only drinking filtered water was significantly associated with lower odds of anti-*Strongyloides* spp. IgG seropositivity in multivariate analysis (AOR = 0.21, 95% CI 0.05–0.95, *p* = 0.043). Children who reported consuming filtered water had a reduced likelihood of seropositivity compared to those drinking tap or rainwater. No statistically significant associations were found for other factors including gender, education level, parental occupation, domestic animal ownership, history of anthelmintic use, handwashing before meals, handwashing after playing, consumption of boiled or filtered water, eating raw or undercooked foods, wearing shoes, or playing on dirt/grass. Observable trends suggested that regular handwashing, shoe use, and not consuming raw food may be associated with lower odds of seropositivity, though these associations did not reach statistical significance (all *p* > 0.05; see Table 3 for full results).

### 3.4. Anti-Strongyloides spp. IgG Seropositivity and Hematological Parameters

One sample was excluded from the analysis of hematological parameters using CBC due to blood clotting, leaving 350 samples for analysis. The results showed that anti-*Strongyloides* spp. IgG-seropositive individuals [median (IQR): 396 (80) × 10^3^/μL] had significantly higher platelet counts than the IgG-seronegative group [median (IQR): 357 (97) × 10^3^/μL] (*p* = 0.042). However, both median values fell well within the normal physiological reference range (140.00–400.00 × 10^3^/μL); therefore, this statistical difference lacks clinical significance. Nevertheless, it is worth noting that both seropositive and seronegative individuals had MCV and MCH values lower than the normal range, indicating microcytic and hypochromic indices suggestive of possible iron deficiency or thalassemia trait, rather than definite anemia, as their hemoglobin and hematocrit levels were generally within normal ranges, as illustrated in Table 4. When examining WBC differentiation, we found that the percentage of eosinophils was higher in the seropositive individuals than in the seronegative individuals (Table 4).

## 4. Discussion

This research provides baseline data on the seroprevalence of anti-*Strongyloides* spp. IgG among primary school children in the island setting of Koh Yao, where access to centralized public health services is limited and no previous epidemiological data have been published. The overall seroprevalence was 3.7%, a rate that contributes valuable insight into the presence of this neglected parasite in a geographically isolated community. The seroprevalence detected in Koh Yao was lower than the pooled regional estimate for anti-*Strongyloides* spp. IgG seropositivity across ten Southeast Asian countries, based on a random-effects model. Our finding also fell below the prevalence levels reported in Thailand’s northeastern region (20.6–61.0%) [24,25] and the southern region, where the average prevalence is approximately 8.3% [8]. By contrast, our finding is greater than the 0.9% seroprevalence noted in a 2017 report from southern Thailand [3]. Several factors may account for these differences. First, variations in diagnostic methods across studies could influence prevalence estimates. The present study used ELISA, a serological method known for its higher sensitivity compared to stool-based techniques, which may have led to improved detection of subclinical or low-intensity infections [26]. Second, temporal factors such as climate change, rainfall, land cover, and other environmental factors may have contributed to changes in prevalence over time [27,28,29].

For sociodemographic analysis, the majority of participants were female, primarily sixth-grade students, and identified as Muslim. A slightly higher proportion of children reported having domestic animals, though the distribution between domestic animal owners and non-owners was relatively even. At this developmental stage, children generally begin practicing self-care and maintaining personal hygiene, behaviors known to reduce the odds of anti-*Strongyloides* spp. IgG seropositivity through decreased exposure to infective larvae. Other observational trends from behavioral analysis indicated that most participants engaged in low-risk practices, which may contribute to the lower observed seroprevalence of anti-*Strongyloides* spp. IgG. This behavior was the consistent use of footwear outdoors. Prior research has established that such hygienic practices are protective against *Strongyloides* spp. exposure [30]. Notably, children who consumed filtered water had significantly lower seropositive rates than those who drank tap or rainwater, likely due to reduced exposure to contaminated sources. This observation is supported by a study in Egypt, where *S. stercoralis* larvae were detected directly in 2.5% (two out of 80 water samples) of tap water samples via microscopy and 5% (four of the 80 water samples) after culture on both non-nutrient agar (NNA) and agar plate culture (APC) [31]. However, this association is borderline and based on a small number of events, making it a hypothesis-generating observation. Since *Strongyloides* spp. is primarily transmitted through skin penetration by infective larvae from contaminated soil rather than through water ingestion, this association should be interpreted with caution. In fact, given the borderline *p*-value, wide confidence interval, and the multiple comparisons performed on a small number of outcome events, this association must be interpreted with extreme caution. Access to filtered water likely serves merely as a proxy marker for better overall household water, sanitation, and hygiene (WASH) practices and higher socioeconomic status, rather than a direct causal protective factor. This may be a potential Type I error due to multiple comparisons [32].

Interestingly, both seropositive and seronegative individuals exhibited eosinophil percentages near the upper limit of the normal reference range. Eosinophilia is a hematological hallmark of parasitic infections [33]. While eosinophilia in the seropositive individuals may reflect a direct response to *Strongyloides* spp. antigens, elevated eosinophil percentages in the seronegative group could result from other etiologies, such as infections with other helminths like *Ascaris lumbricoides* or hookworm, allergic diseases, or other underlying conditions [34,35,36]. A recent study reported that approximately 5% of children in the Koh Yao Islands were infected with soil-transmitted helminths, which may also contribute to the eosinophilia observed among schoolchildren in this study [18]. Platelet counts were slightly higher in the seropositive individuals than in the seronegative individuals; however, despite statistical significance, the difference was not clinically relevant. The clinical relevance of increased platelet counts in anti-*Strongyloides* spp. IgG seropositive children remain uncertain. Previous studies have not established a direct relationship between strongyloidiasis and thrombocytosis in otherwise healthy individuals. The finding in our cohort may reflect random variation due to the small number of seropositive cases, rather than a true pathogenetic effect. Further research, involving larger sample sizes and inclusion of clinical outcome data, is necessary to determine whether platelets could serve as a marker of seropositivity or merely represent coincidental variability [37].

This study has several important limitations. First, the cross-sectional design limits causal inferences. Additionally, participation depended on voluntary consent, which inherently introduces potential selection bias, meaning the sample may not perfectly represent the total population. Second, the study relied exclusively on ELISA-based serology, which detects IgG antibodies. This method cannot reliably distinguish between past, resolved exposures and active current infections, and it is subject to potential cross-reactivity with other soil-transmitted helminths. Third, the multivariate logistic regression analysis was based on a very low number of seropositive cases (n = 13), resulting in a low events-per-variable ratio. Consequently, there is a high risk of model overfitting. Therefore, this multivariable risk-factor analysis is strictly exploratory, and the adjusted odds ratios must be interpreted with extreme caution as hypothesis-generating findings rather than confirmatory evidence. Moreover, most behavioral and environmental variables exhibited extremely low variability (e.g., >90% of participants reporting the same practices). This near-homogeneity compromises the logistic regression model’s ability to reliably detect associations, further underscoring the need to interpret the odds ratios with caution. Fourth, behavioral risk factors were collected via self-reported, interviewer-administered questionnaires, which are inherently subject to recall bias and social desirability bias. Fifth, data collection was conducted solely during the dry season; seasonal variations, such as increased soil moisture during the rainy season, could influence transmission dynamics. Sixth, the sample size calculation was based on adult seroprevalence data. Furthermore, the exclusion of immunocompromised children may have systematically reduced the probability of detecting seropositive individuals. Finally, the findings from school-aged children in a single island setting cannot be generalized to adults, mainland populations, or immunocompromised individuals.

## 5. Conclusions

Our findings provide serological evidence of anti-*Strongyloides* spp. IgG seropositivity among primary school children in Koh Yao, Phang Nga Province, southern Thailand. The observed low seroprevalence highlights the relatively limited transmission in this rural island community but also emphasizes the need for continued vigilance. While a borderline association with filtered water consumption was observed, it likely reflects broader household hygiene practices rather than a direct protective factor. The findings underscore the importance of raising awareness among local schoolchildren and the broader population regarding parasitic infections. By offering area-specific data and identifying environmental and household factors, this study supports the development of targeted prevention and control strategies tailored to island or remote regions of Thailand. Further longitudinal and molecular studies are recommended to monitor transmission dynamics, validate serological findings, and explore the impact of co-infections and environmental factors on disease risk.

## Figures and Tables

**Table 1:** Sociodemographic characteristics among primary school children in Koh Yao, Phang Nga Province.

Group	Number	%
**Total**	351	
**Gender**		
Male	156	44.4
Female	195	55.6
**Age (years)** (median (IQR))	11 (2)	
**Education level**		
Grade 1	2	0.57
Grade 2	37	10.54
Grade 3	42	11.97
Grade 4	58	16.52
Grade 5	88	25.07
Grade 6	124	35.33
**Father’s or Mother’s occupation**		
Not Agricultural worker	324	92.31
Agricultural worker	27	7.69
**Have domestic animals**		
No	141	40.17
Yes	210	59.83
**Anthelmintic drug**		
No	171	48.72
Yes	180	51.28
**Wash your hands before meal**		
No	5	1.42
Yes	346	98.58
**Wash your hands after playing**		
No	19	5.41
Yes	332	94.59
**Drinking water source**		
Tap/Rainwater	31	8.83
Water from the filter	320	91.17
**Water you drink, boiled or filtered**		
No	40	11.40
Yes	311	88.60
**Eating raw or undercooked food**		
No	208	59.26
Yes	143	40.74
**Wearing shoes**		
No	25	7.12
Yes	326	92.88
**Play on dirt or grass**		
No	10	2.85
Yes	341	97.15

**Table 2:** Seroprevalence of anti-*Strongyloides* spp. IgG among 351 students from 12 primary schools in Koh Yao, Phang Nga Province.

Study Site ^1^		N	Number of Seropositive (%)	95% CI	*p*-Value ^2^
**Koh Yao Noi**					0.872
	Sc1	19	1 (5.3)		
	Sc2	27	0 (0.0)		
	Sc3	68	4 (5.9)		
	Sc4	26	1 (3.8)		
**Total**		140	6 (4.3)	1.81, 8.62	
**Koh Yao Yai**					
	Sc5	60	2 (3.3)		
	Sc6	10	1 (10.0)		
	Sc7	30	0 (0.0)		
**Total**		100	3 (3.0)	0.85, 8.89	
**Phru Nai**					
	Sc8	32	1 (3.1)		
	Sc9	27	0 (0.0)		
	Sc10	32	1 (3.1)		
	Sc11	10	0 (0.0)		
	Sc12	10	2 (20.0)		
**Total**		111	4 (3.6)	1.23, 8.34	
**Overall**		**351**	**13 (3.7)**	**2.09, 6.07**	

^1^ Abbreviation Sc = School, ^2^ Data analysis by chi-square test.

**Table 3:** Factors associated with anti-*Strongyloides* spp. IgG seropositivity among primary school children in Koh Yao, Phang Nga Province.

Characteristics ^3^		N (%)	*Strongyloides* spp. Seropositivity (*n*/N (%))	OR (95% CI)	*p*-Value	AOR (95% CI)	*p*-Value
**Gender**							
	Male	156 (44.44)	3/156 (1.92)	Ref. ^1^	0.129	Ref. ^1^	0.121
	Female	195 (55.56)	10/195 (5.13)	2.76 (0.75–10.20)		2.93 (0.75–11.42)	
**Education level**							
	≤Grade 3	81 (23.08)	3/81 (3.70)	Ref. ^1^	1.000	Ref.^1^	0.744
	>Grade 3	270 (76.92)	10/270 (3.70)	1.00 (0.27–3.72)		0.78 (0.17–3.41)	
**Father or Mother’s occupation**							
	Not Agricultural worker	324 (92.31)	12/324 (3.70)	Ref. ^1^	1.000	Ref. ^1^	0.785
	Agricultural worker	27 (7.69)	1/27 (3.70)	1.00 (0.13–7.99)		1.37 (0.15–12.78)	
**Have animals**							
	No	141 (40.17)	6/141 (4.26)	Ref. ^1^	0.655	Ref. ^1^	0.373
	Yes	210 (59.83)	7/210 (3.33)	0.77 (0.26–2.36)		0.582 (0.15–1.74)	
**Anthelmintic drug**							
	No	171 (48.72)	8/171 (4.68)	Ref. ^1^	0.351	Ref. ^1^	0.287
	Yes	180 (51.28)	5/180 (2.78)	0.58 (0.19–1.82)		0.52 (0.15–1.74)	
**Wash your hands before meal**							
	No	5 (1.42)	1/5 (20.00)	Ref. ^1^	0.093	Ref. ^1^	0.242
	Yes	346 (98.58)	12/346 (3.47)	0.14 (0.02–1.39)		0.18 (0.01–3.18)	
**Wash your hands after playing**							
	No	19 (5.41)	1/19 (5.26)	Ref. ^1^	0.713	Ref. ^1^	0.485
	Yes	332 (94.59)	12/332 (3.61)	0.71 (0.08–5.48)		0.45 (0.05–4.30)	
**Drinking water source**							
	Tap/Rainwater	31 (8.83)	3/31 (9.68)	Ref. ^1^	0.081	Ref. ^1^	0.043 *
	Filtered water	320 (91.17)	10/320 (3.13)	0.30 (0.08–1.16)		0.21 (0.05–0.95)	.
**Drink boiled or filtered water** ^2^							
	No	40 (11.40)	1/40 (2.50)	Ref. ^1^	0.671	Ref. ^1^	0.295
	Yes	311 (88.60)	12/311 (3.86)	1.57 (0.20–12.37)		3.44 (0.34–34.80)	
**Eating raw or undercooked food**							
	No	208 (59.26)	9/208 (4.33)	Ref. ^1^	0.459	Ref. ^1^	0.754
	Yes	143 (40.74)	4/143 (2.80)	0.64 (0.19–2.11)		0.81 (0.22–2.97)	
**Wearing shoes**							
	No	25 (7.12)	2/25 (8.00)	Ref. ^1^	0.253	Ref. ^1^	0.849
	Yes	326 (92.88)	11/326 (3.37)	0.40 (0.08–1.92)		0.51 (0.07–3.39)	
**Play on dirt or grass**							
	No	10 (2.85)	1/10 (10.00)	Ref. ^1^	0.309	Ref. ^1^	0.359
	Yes	341 (97.15)	12/341 (3.52)	0.33 (0.04–2.80)		0.35 (0.03–3.31)	

^1^ Ref. refers to the reference category in categorical variables. * Statistically significant at *p*  <  0.05; OR: odds ratio by univariate analysis; AOR: adjusted odds ratio by multivariate analysis; CI: 95% confidence interval. ^2^ “Drink boiled or filtered water” was assessed as a dichotomous variable (yes = participant typically consumes water that is boiled or run through a filter before consumption; no = otherwise). This is distinct from “drinking water source,” which categorized the primary source of water (e.g., tap, rainwater, filtered system). While the yes/no dichotomy allows for efficient data capture in field surveys, it may not fully differentiate mixed or intermittent practices. Future questionnaires should consider including multiple-choice or frequency-based responses to improve measurement precision. ^3^ The question on shoe-wearing behavior (“How often do you wear shoes?”) and other similar items were initially measured using a Likert-type scale with three response categories: always, sometimes, and never. For analysis and table presentation, these were dichotomized as “consistent use” (“always”) versus “inconsistent use” (“sometimes” or “never”), primarily to address small cell sizes and facilitate epidemiological comparison. We recognize that this dichotomy may overlook variation in actual behavior and recommend that future studies analyze such variables as ordinal categories to provide more nuanced information.

**Table 4:** Comparison of the hematological parameters between anti-*Strongyloides* spp. IgG seropositive individuals (n = 13) and seronegative individuals (n = 337).

Blood Parameters	Normal Ranges	Groups		*p*-Value ^2^
		Seropositive ^1^ (n = 13)	Seronegative ^1^ (n = 337)	
RBC (×10^6^/µL)	3.80–5.80	4.86 (0.61)	4.83 (0.52)	0.722
Hemoglobin (g/dL)	11.00–16.50	12.40 (1.40)	12.50 (1.10)	0.574
Hematocrit (%)	35.00–50.00	37.10 (2.90)	37.60 (3.00)	0.277
MCV (fL)	80.00–98.00	77.30 (7.90)	78.80 (7.20)	0.362
MCH (pg)	27.00–32.00	26.40 (2.80)	26.30 (3.10)	0.708
MCHC (g/dL)	32.00–36.00	33.20 (1.40)	33.30 (1.20)	0.963
RDW (%)	11.50–14.00	13.10 (1.40)	12.90 (1.20)	0.247
WBC (×10^3^/µL)	5.00–10.00	8.14 (2.96)	8.06 (2.56)	0.573
Neutrophils (%)	40.00–80.00	42.30 (14.80)	46.30 (14.80)	0.647
Lymphocytes (%)	20.00–70.00	46.20 (13.70)	42.00 (14.50)	0.506
Eosinophils (%)	0.00–6.00	3.10 (3.50)	3.00 (3.60)	0.635
Monocytes (%)	2.00–10.00	6.70 (1.70)	6.60 (2.00)	0.991
Basophils (%)	0.00–2.00	0.50 (0.40)	0.50 (0.30)	0.513
Platelet (×10^3^/µL)	140.00–400.00	396.00 (80.00)	357.00 (97.00)	0.042 *

^1^ Data were expressed as median (IQR), ^2^ Data analysis by Mann–Whitney U; * Significant at *p*-value < 0.05.

## Data Availability

The data associated with this study has been included in this article. Further inquiries can be directed to the corresponding author.
